# Development of a sequential laser microdissection tissue cuts workflow for the spatial and quantitative analysis of drugs in fresh frozen tissue sections

**DOI:** 10.1371/journal.pone.0312542

**Published:** 2024-12-23

**Authors:** Elias Maris, Farid Jahouh, Kathleen Allaerts, Rob Vreeken

**Affiliations:** 1 Department of Bioanalysis, Ghent University, Ghent, Belgium; 2 Johnson & Johnson, Beerse, Belgium; 3 Faculty of Health, Maastricht MultiModal Molecular Imaging Institute (M4I), Medicine and Life Sciences, Maastricht University, Maastricht, The Netherlands; 4 TNO, Healthy Living & Work, Leiden, The Netherlands; Zhejiang Cancer Hospital, CHINA

## Abstract

Mass spectrometry imaging (MSI) is a well-established technique that allows to determine the distribution of small molecules, such as lipids, metabolites, and drugs, as well as large molecules in tissue sections. Because of the tissue heterogeneity, resulting in different matrix effects, and to the fact that the measured compounds are not entirely “extracted” from the tissue during the measurement, the absolute quantitative aspect of MSI is limited. To combine compound quantification with spatial information on fresh frozen unstained tissue sections, laser (capture) microdissection has been used to isolate tissue sections for compound extraction and LC-MS/MS quantification. Although this method relying on manual ROIs selection is rather sensitive compared to traditional MSI methods, it lacks the throughput needed to screen entire tissue sections. To apply a higher throughput tissue screening approach, we propose herein a workflow for performing indiscriminate and sequential LMD tissue section cuts that can cover up to 96 cuts collected in a 96 well plate on Leica LMD systems, for further extractions and LC-MS/MS analysis. Our workflow relies on the creation and implementation of 96 squares microgrid templates for the LMD cut of different area sizes (30x30 μm^2^, 50x50 μm^2^, 100x100 μm^2^, 200x200 μm^2^, 270x270 μm^2^ and 500x500 μm^2^) using 5 different magnifications (5x, 10x, 20x, 40x and 63x), on fresh frozen tissue sections. The method was applied on 20μm mouse brain and liver tissue sections. The tissue cut collection yields were evaluated visually and by the detection of the sprayed standards on the tissue sections, and found to be ranging from 78% to 91%, and the throughput of the LMD cuts and collection in a 96 well format, was measured to be from 19 to 37 minutes per tissue section, depending on the 96 squares microgrid template and the corresponding magnification lens used. Further extraction and LC-MS/MS analysis of 3 different compounds previously sprayed on a mouse liver tissue section allowed to determine the LLOQ the workflow allows to achieve when using the different templates.

## Introduction

In drug discovery and development, the *in vivo* drug distribution and quantification after its administration is fundamental in understanding its pharmacological and toxicological effect [1–3]. In bioanalysis, liquid chromatography coupled to tandem mass spectrometry is the most widely used technique for the quantification of drugs in tissue homogenates, because of its sensitivity, selectivity and versatility towards a large class of molecules [4,5]. However, this method lacks spatial information.

Autoradiography (ARG) is one of the standard methods within preclinical studies to determine and quantify pharmaceutical compounds tissue distribution [6]. Despite its high sensitivity, this technique is not selective enough to discriminate the parent drug and its different metabolites, relies on the availability of radiolabeled compounds, and is rather costly. The emergence of Mass Spectrometry Imaging (MSI) techniques allowed to overcome some technical limitations of ARG, since it is a label free imaging technique, and its selectivity enables to discriminate the parent drug distribution from its metabolites. Matrix Assisted Laser Desorption Ionization- (MALDI) and Desorption Electrospray Ionization- (DESI) are the most widely used ionization sources for MSI [7]. Although MSI is a powerful technique to obtain spatial information of the compounds on tissue sections, its sensitivity towards low tissue drug concentration is limited due to the high ionization suppression the compounds encounter [8]. The absolute quantification aspect of MSI is rather limited, by the restricted availability of stable isotopically labeled internal standards for each measured compound to correct for the ionization suppression in the different tissue regions [9]. The compounds extraction yields from the tissue during MALDI- and DESI-MSI is also a limiting factor for absolute quantification during MSI experiments [9,10].

Laser (capture) MicroDissection (LMD) is another technique used to combine spatial information with molecular tissue composition [11–13]. LMD has been widely used for the dissection and isolation of specific cells and regions of interest (ROIs) from different tissues, for analysis of nucleic acids and proteins, in formalin fixed paraffin embedded (FFPE) and cryopreserved tissue [14].

More recently, LMD has been applied on fresh frozen tissue sections in combination with LC-MS/MS for small and large molecules quantification on specific tissue regions [15–17]. This approach allows to combine the spatial information of the LMD to the sensitivity and selectivity of LC-MS/MS. However, this workflow presents some technical challenges when applied for the quantification of small molecules. In fact, this approach can only be applied on fresh frozen tissue sections, since during the tissue fixation and embedding with FFPE, the small molecules can be washed out of the tissue, and the presence of paraffin introduces contamination during the sample extraction after LMD. Another challenge is the inability to stain the tissue section before LMD (with H&E for example), because of washing away the analytes during this procedure. The fact that the LMD cuts are guided by an optical image of an unstained tissue section makes it challenging to review ‘histo-regions’. In these conditions, without proper guidance, compound tissue exposure can be achieved by performing sequentially LMD biopsies on the entire tissue, followed by LC-MS/MS.

In this study, we developed a method for the sequential isolation and capture of squared tissue areas, collected in a 96 well plate, aiming to screen the entire tissue section. Templates of different tissue cut sizes (30x30 μm^2^ to 500x500 μm^2^), consisting of an array of squares arranged in 12 lines of 8 rows, have been developed on a Leica LMD system and are made available in this manuscript. This method is aimed to be used with LC-MS/MS for absolute quantification of compounds in the tissue sections. Parameters such as tissue thickness, tissue drying time, cut sizes (30x30 μm^2^ to 500x500 μm^2^) and laser power were evaluated and optimized in different tissue types. Three test compounds will be sprayed on top of the tissue section before LMD in order to evaluate the extraction reproducibility throughout the different tissue cuts.

## Materials and methods

### Reagents

The following compounds, selected based on their physico-chemical differences were used for the experiments and obtained from a Janssen compound library: darunavir, loperamide and a Janssen (JNJ) compound.

All solvents used in the LC-MS/MS method are UPLC-grade. Methanol (MeOH), formic acid (FA) and dimethyl sulfoxide (DMSO) was obtained from Merck KGaA (Darmstadt, Germany). Trifluoroacetic acid (TFA) was obtained from Sigma-Aldrich (St. Louis, MO, USA). Isopropanol (IPA) was obtained from VWR International (Radnor, PA, USA). Acetonitrile (ACN) was purchased from Biosolve Chimie (Dieuze, France). Milli-Q water was obtained from a Milli-Q Advantage A10 Water Purification system from Millipore (Burlington, MA, USA). A H&E staining kit was purchased from Abcam (Cambridge, UK) and included hematoxylin (modified Mayer’s solution), Eosin Y solution, and bluing reagent. Dulbecco’s Phosphate Buffered Saline (DPBS) was purchased from Sigma-Aldrich (St. Louis, MO, USA) Paraformaldehyde (PFA) solution (4% in PBS) was obtained from Thermo Fisher Scientific (Waltham, MA, USA).

### Tissue preparation

Blank tissue liver and brain from mice (Balb/c, male, supplied by Janvier) and rats (Wistar Han, male, supplied by Charles River Germany) were obtained from the Janssen or JnJ *in vivo* Sciences department and stored at a temperature of -80°C before sectioning.

#### Tissue cryosectioning

Cryosectioning was performed using a Leica CM3050 S Research cryostat (Leica Biosystems, Nussloch, Germany). The frozen organs were first mounted on the tissue holder by applying enough water to cover the surface of the tissue holder before placing the organ on top of it inside the cryostat chamber. Once the water was frozen and the organ sticked to the tissue holder, the sectioning was carried out at object (OT) and chamber (CT) temperatures of -17°C for liver tissue and -16°C for brain tissue. Tissue sections of different thicknesses (12–200μm) were thaw-mounted on 4.0μm polyethylene naphtalate (PEN) membrane slides (#11600289, Leica Microsystems, Wetzlar, Germany) and dried in a desiccator before being stored at -80°C until the LMD experiment.

#### Tissue spraying with compounds of interest

An HTX M5 Sprayer (HTXImaging, by HTX Technologies, Chapel Hill, NC) was used to spray a mixture of 3 compounds, i.e loperamide, dorunavir and a JNJ compound, prepared at a concentration of 10 μg/mL in methanol containing 0.1% TFA on the tissue sections, under the following conditions: 16 passes (CC pattern) at a flow rate of 0.2 mL/min, a nozzle velocity of 1200mm/min, a nozzle temperature of 70°C, and a tracking space of 3mm.

### Laser microdissection

The LMD experiments were conducted using a Leica LMD7 system (Leica Microsystems, Wetzlar, Germany) operated by the Leica Laser Microdissection V8.3 software that allowed us to create and implement the 96 squares microgrids. Currently, with the new release from Leica, the templates can be uploaded and implanted in the Leica Microdissection V8.4 version. The tissue sections were dried in a vacuum desiccator for 15 min to 1.5 hours, followed by the microdissection. Using the middle pulse option, a final laser pulse in the middle of the dissected area is used to drop this inside the collector. The tissue cuts were collected in a sterile, non-pyrogenic, flat bottom, ultra-low attachment surface polystyrene 96-well plate with lid (Ref 3474, Corning Costar®, Kennebunk, ME, USA).

#### 96 squares microgrid templates development

The 96 squares microgrid templates were developed with the help of Leica Microsystems. With the aid of microscopy image analysis software AIVIA (Leica Microsystems, Bellevue, WA), an importable.xml file containing a non-fixed template and adaptable X and Y calibration points for a 96-squares microgrid was created.

#### Compound tissue extraction

Extraction was done directly after LMD using 100μL MeOH 0.1% TFA. Hereafter, the 96-well collection plate was sonicated in a water bath for 10 minutes at room temperature. To correct for variations in instrument performance, and measurement conditions, the internal standard verapamil is spiked into the extraction solvent at a concentration of 10 ng/mL as an internal standard.

### LC-MS/MS

Liquid Chromatography was performed using a Waters Acquity UPLC I-class system (Milford, MA, USA). Chromatographic separation was performed on an ACQUITY UPLC® BEH-C18 column (50mm×1mm, 1.7μm) using a gradient elution of H_2_O 0.1% FA (mobile phase A, 98%) and acetonitrile (mobile phase B, 2%), both containing 0.1% formic acid and at a flow rate of 0.2mL/min. The gradient profile consisted of an initial condition of 2% mobile phase B for 1minute, followed by a linear decrease to 98% of B over 3 minutes.

Mass spectrometry analysis was carried out on a Waters Xevo-TQ-S triple quadrupole mass spectrometer (Milford, MA, USA) with an ESI probe operating in positive ionization mode. Nitrogen was used as desolvation and nebulization gas and argon was used as collision gas. The detailed mass spectrometer operating conditions were as follows: a desolvation gas-flow of 790 L/hr, a desolvation temperature of 350°C, a source temperature of 150°C, a cone gas-flow of 150 L/hr, a nebulizer gas flow of 5.78 Bar, a collision gas-flow of 0.14 mL/min and a capillary voltage of 3.6 kV. The MRM transitions of the compounds, the cone voltage, collision energy and dwell time are described in Table 1.

**Table 1:** Tuning parameters and linear range of the different standards used in the LC-MS/MS experiment.

	*Precursor ion m/z*	*Product ion m/z*	*Cone voltage (V)*	*Collision Energy (eV)*	*Linear range (ng/mL)*
***JNJ compound***	-	-	-	-	0.1–500
***Darunavir***	548.3	392.2	14	16	0.1–2000
	548.3	140.7	14	36	
***Loperamide***	477.3	266.2	14	24	0.1–500
	477.3	210.2	14	46	
***Verapamil (IS)***	455.3	165.0	50	40	-

## Results & discussion

**Fig 1:**
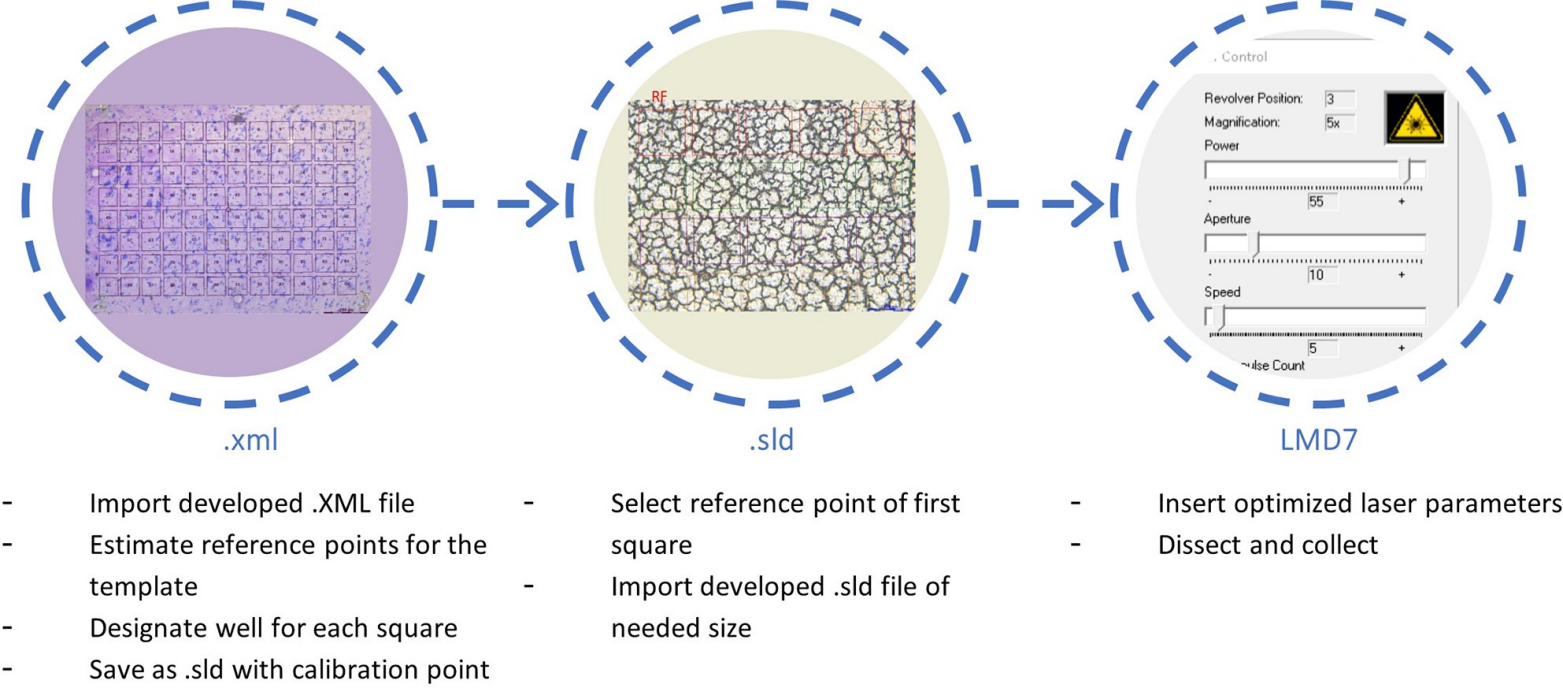
Workflow for the development of an importable 96 squares microgrid format for the LMD7 software, proposed by Leica microsystems.

In this work, we present the development and application of 96 squares microgrids for the sequential laser microdissection of fresh frozen tissue sections for further compounds extraction and LC-MS/MS quantification (Fig 1).

Parameters the tissue thickness, the magnification lens used during the experiment and cutting laser conditions have been determined for the LMD experiment [18–21].

### Tissue thickness influence on the LMD cuts

**Fig 2:**
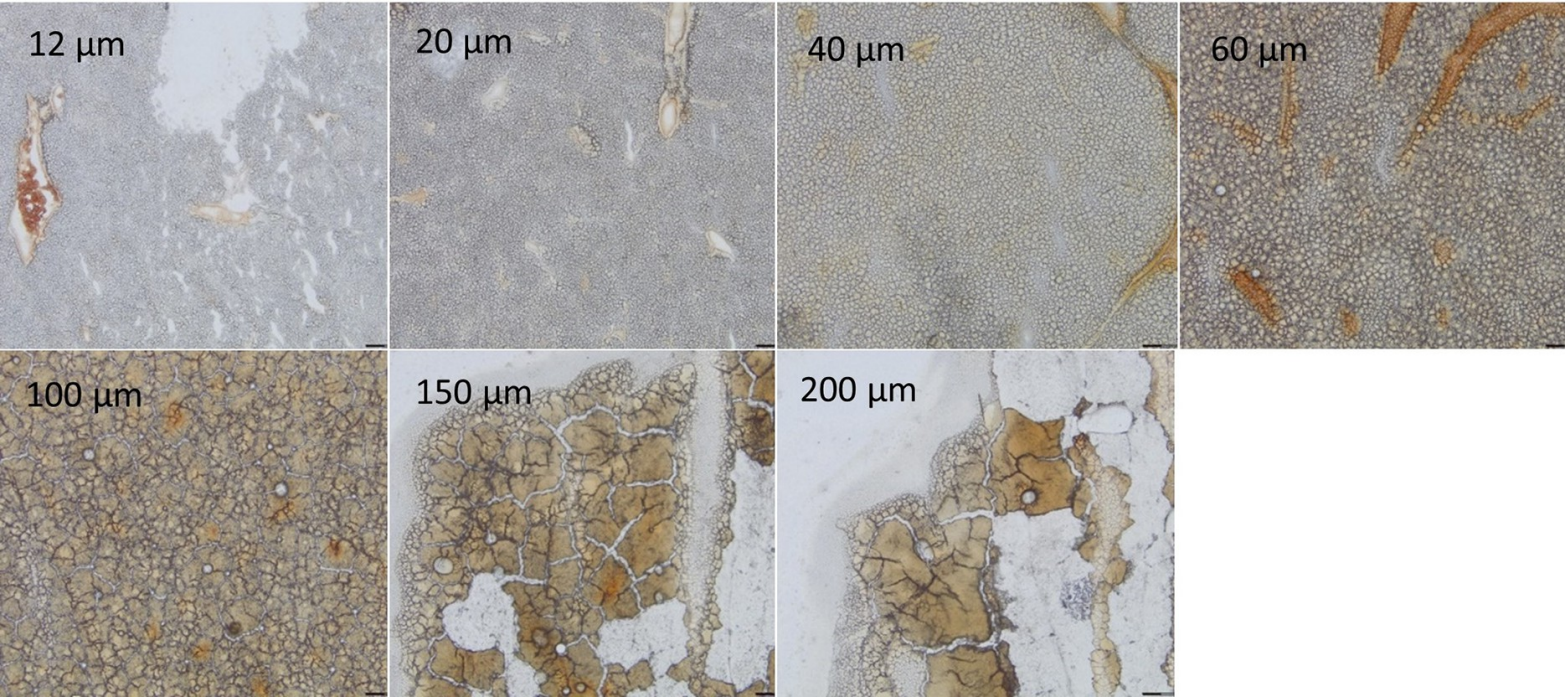
Images of fresh frozen liver tissue sections of varying thicknesses ranging from 12 to 200 μm, mounted on PEN membrane slides, to evaluate the tissue homogeneity and morphology. Captured with a x2.5 objective.

The volume of the material collected during LMD depends on the area of the dissection and the thickness of the tissue. In view of maximizing the collected material volume, we optimized the tissue thickness (12–200 μm) during the sectioning of a mouse liver tissue (Fig 2). Hence, tissue sections within the range of 20–100 μm in thickness showed to have adequate quality for dissection (Fig 2). Above 100 μm in thickness, sections showed cracks in the tissue and the tissue mounting on the PEN membrane is more challenging. Moreover, we found it challenging to cut through a tissue of 200 μm in thickness since it requires a high laser power and does burn much more tissue compared to lower tissue thicknesses. Below 20 μm in thickness, we observed some heterogeneity of the tissue on the PEN frame, corresponding to areas that contain tissue and empty areas. Samples with thicknesses ranging from 20 to 60 μm provided the best results in terms of tissue morphology and LMD applicability.

### Tissue section drying time

Tissue moisture has a significant influence on the LMD experiment of frozen tissue sections. In fact, moisture in the tissues makes it more challenging to cut during LMD and a dried tissue section can be subjected to electrostatic effect during the LMD experiment, both effects minimizing the tissue cuts capture efficiency in the “cut and drop” LMD system.

Before the LMD experiment, the tissue sections were dried in a vacuum desiccator. Because of potential on-tissue enzymatic activity that can lead to compounds degradation, a tissue drying time of 30 minutes is recommended.

### 96 squares microgrid templates development

Another parameter to consider for the LMD experiment is the choice of the magnification lens that can influence the tissue surface loss during cutting. In fact, the higher the magnification, the smaller the laser beam size. During our experiment, the laser beam size was measured at different magnifications on the PEN frame and using the optimized laser parameters for each magnification. We observed the following laser beam sizes at the different magnifications: 7.8 μm (5x), 4.5 μm (10x), 2.6 μm (20x), 1.8 μm (40x) and 0.8 μm (63x). On a 20 μm mouse liver tissue section, the laser beam size was evaluated to be for the different magnifications: 9.1 μm (5x), 5.2 μm (10x), 3.6 μm (20x), 2.1 μm (40x), 1.1 μm (63x) (Table 2). Hence, if the 96 squares microgrid is applied in the field view only, it will result in a higher volume of tissue loss during the cutting compared to the same cut size at a higher magnification. This is why we developed a 96 squares microgrid that is deployed partly inside and partly outside of the field view. On top of that, the tissue cut collection is automatically assigned to different wells of a 96-well plate collector.

**Table 2:** Characteristics of the different 96 squares templates: Average single square area (μm^2^), relative bridge distance (%), cutting time (min) and laser tissue cutting width (μm) determined on a 20μm thick fresh frozen mouse liver tissue section.

*Template (μm × μm)*	*Magnification lens*	*Average area (μm* ^*2*^ *)*	*Relative bridge distance (%)*	*Cutting Time (min)*	*Laser tissue cutting width (μm)*
***500 x 500***	x5	246363	12	32	9.1 (1.8%)
***270 x 270***	x10	73483	15	19	5.2 (1.9%)
***200 x 200***	x20	39969	13	37	3.6 (1.8%)
***100 x 100***	x40	10134	8	20	2.1 (2.1%)
***50 x 50***	x63	2563	10	29	1.1 (2.2%)
***30 x 30***	x63	923	13	24	1.1 (3.7%)

The development of the 96-square microgrid templates is described in Fig 1. An.xml file containing the coordinates of the 96 squares distributed in 8 lines and 12 columns without an assigned size was first created using the AIVIA software. After the estimation of the reference points to achieve the desired size and shape of the 96-square microgrid, the.xml file was imported in the Leica Laser Microdissection V8.4 software, and a collection well for each cut was assigned to cover a 96-well plate. The template is then saved in.sld format that contains the coordinates of the squares as well as their collection wells in a 96-well plate. In this.sld format, new reference points (square 1: row 1, column 1) are introduced to facilitate the free choice of insertion of the 96-square microgrid over the tissue area of interest. Next to this, the optimal cutting objective for each template size is included as well. Templates were generated with the following square sizes, at different magnifications (Table 2): 30×30 μm^2^ (magnification of 63x), 50×50 μm^2^ (magnification of 63x), 100×100 μm^2^ (magnification of 40x), 200×200 μm^2^ (magnification of 20x), 270×270 μm^2^ (magnification of 10x) and 500×500 μm^2^ (magnification of 5x). Table 2 describes the characteristics of the different 96 squares microgrid templates implemented on a 20 μm thick fresh frozen mouse liver tissue section: average single square area (μm^2^), relative bridge distance (%), cutting time (min) and laser tissue cutting width (μm).

**Fig 3:**
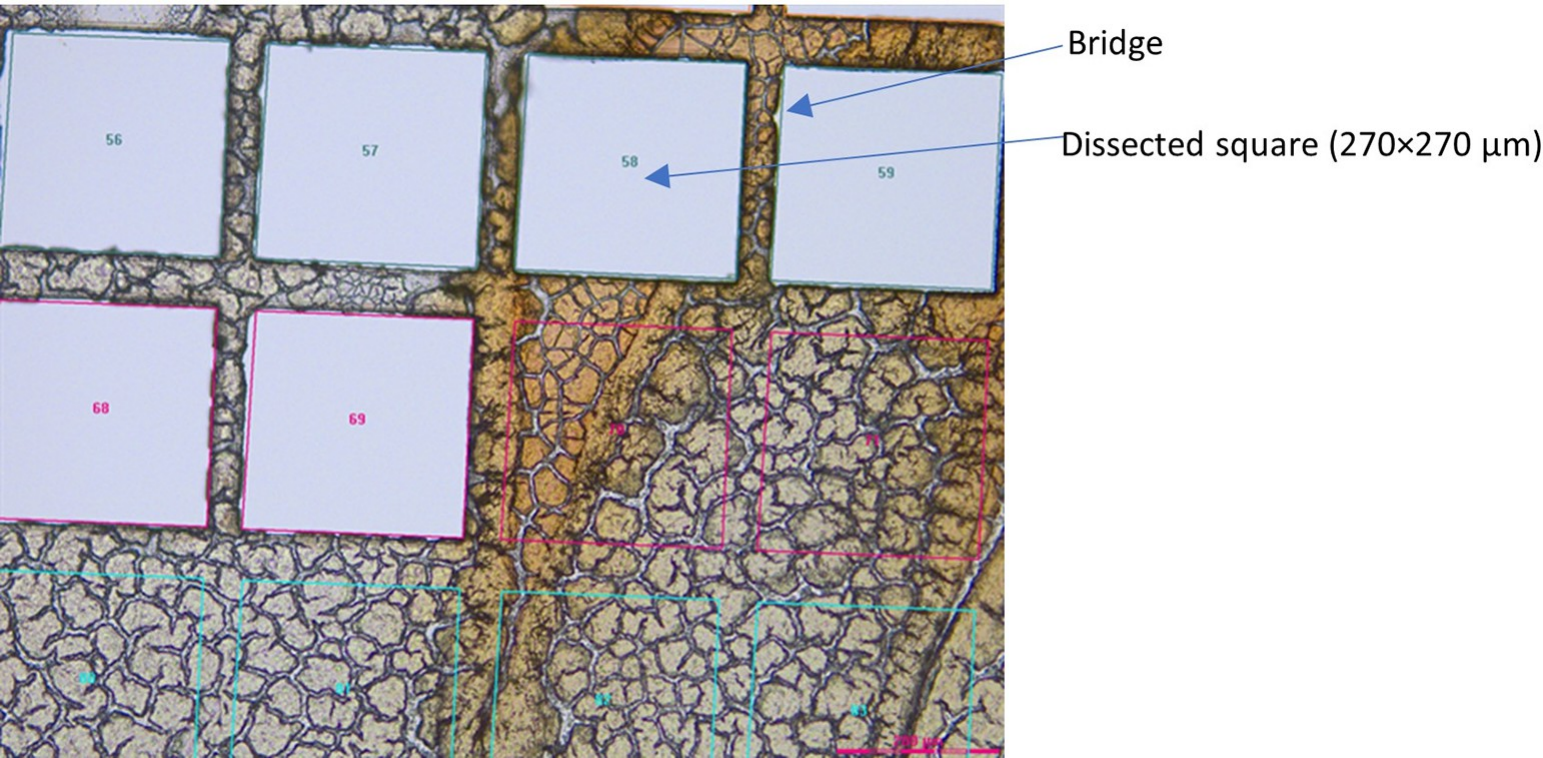
Overview of a partly dissected tissue section using the 270×270 μm 96 squares microgrid template on a fresh frozen mouse liver tissue section (x10 magnification).

The insertion of the template on the tissue section view was carried out by importing the (S1–S6 Files) of the desired size and at the desired magnification (File -> import shapes). A reference point for the first square (column 1, row 1) is then selected on the field view of the tissue and a second reference point that should be horizontal to the first one and at a distance covering the first line of the microgrid is selected. The templates are also provided as (S7–S12 Files) that can be converted to.sld files before being used (renaming the file with a.txt format). Once the template is implemented on the tissue view, slight positional changes can be made after the selection of the whole 96-square grid, whereafter the cuts can be started sequentially and collected in the assigned wells of the 96-well plate. The total tissue cut and collection time was evaluated to be 19 to 32 minutes, depending on the template used (Table 2). Fig 3 displays an area of a liver tissue with consecutive LMD cuts performed using a 270×270 μm^2^ 96 squares microgrid template with a 10x magnification lens. As mentioned in the image, the bridge width, which consists of the distance between the squares is important to keep the integrity of the remaining tissue between the squares (Fig 3). Without bridges, part of the mounted tissue could collapse which makes subsequent dissection impossible with the same laser settings. The bridge distance should be kept to a minimum to minimize loss of data due to these remaining pieces of tissue. As shown in Table 2, the size of the bridge relative to the squares sizes was kept the same for the different templates since it was found to be optimal for the squares cutting and the preservation of tissue integrity after each cut. More specifically, the width of the bridge was on average 12% of the side of the neighboring squares (relative bridge distance). The laser tissue cutting width, corresponding to the laser beam size measured on tissue, was evaluated for the different templates and varies from 1.8% to 3.7%, depending on the selected microgrid (Table 2). It is also important to realize that since the tissue cutting parameters, such as laser power, aperture and frequency are also impacted by the tissue type and thickness, the reported characteristics (relative bridge distance, laser tissue cutting width and cutting time) of the templates might vary when using different tissue thicknesses. Additionally, for fresh frozen dried liver tissue, the laser is only able to cut through a thickness of 150 μm with a 5x magnification lens (template 500×500 μm^2^). For a thickness of 60 and 100 μm, the laser is not powerful enough for cutting at a 63x magnification objective (template 50×50 μm^2^ and 30×30 μm^2^) while leaving a bridge between consecutive cuts.

**Fig 4:**
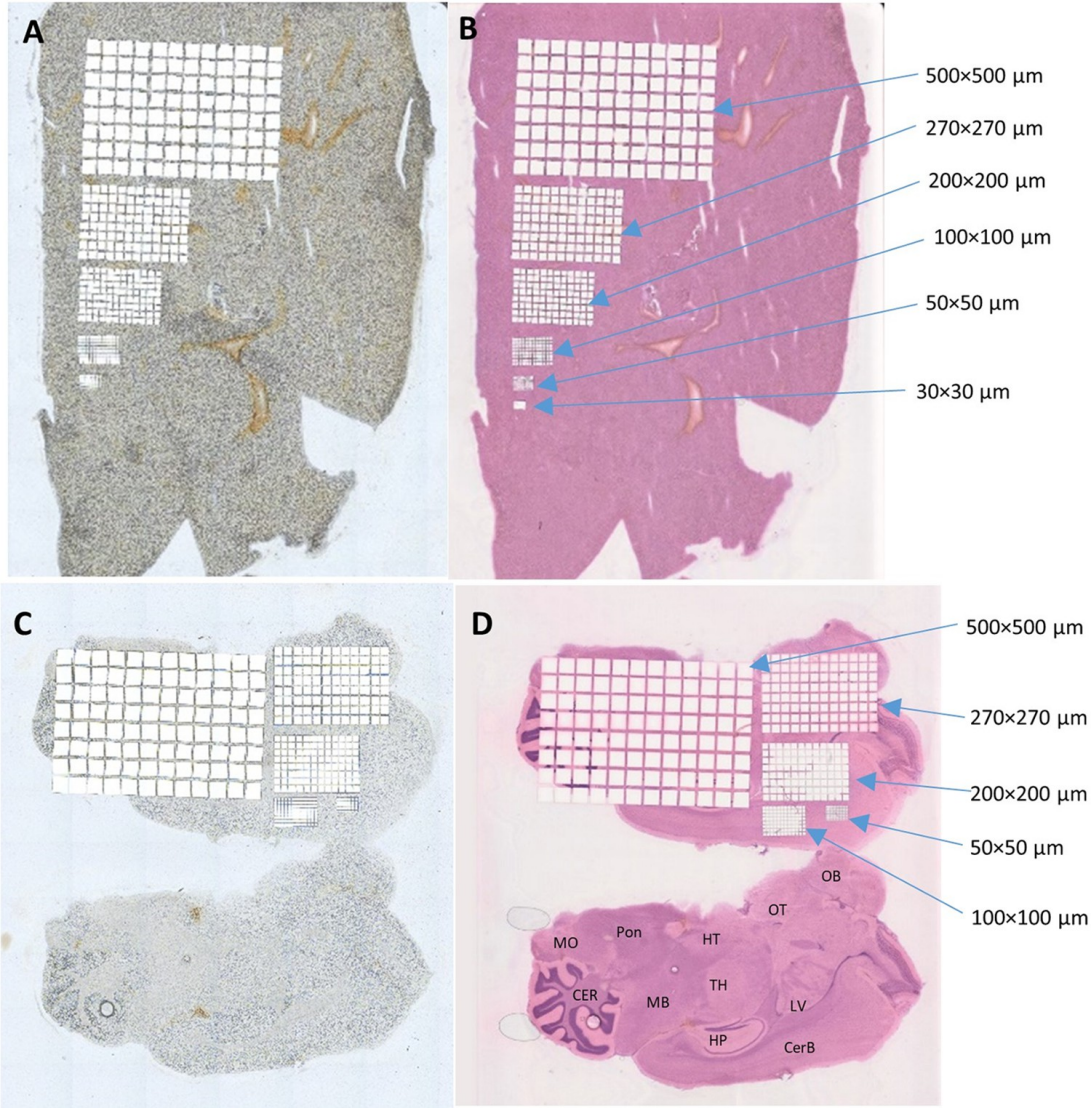
Dissected 96-well formats for all templates (30, 50, 100, 200, 270, 500 μm) with optimized laser and microscope settings. (A) Fresh frozen mouse liver section. (B) Fresh frozen liver section after H&E staining. (C) Fresh frozen mouse brain tissue section. (D) Fresh frozen right brain section after H&E staining. Abbreviations used are as follows: *CER = cerebellum; CerB = cerebrum; HP = hippocampus; HT = hypothalamus; LV = lateral ventricle; MB = midbrain; MO = medulla oblongata; OB = olfactory bulb; OT = olfactory tubercle and TH = thalamus* [22].

Overviews of H&E stained and unstained mouse liver (A and B) and brain (C-D) tissue sections after LMD using the different microgrid sizes are displayed in Fig 4. The 96 squares microgrids of different sizes allow to cover different tissue surface, and thus depending on the spatial resolution and tissue region of interest, the microgrid with the appropriate size can be used. Due to the heterogeneity of the tissues and to the destructive aspect of the LMD, we usually perform H&E staining on the dissected section after LMD and also on an adjacent tissue section. The overlay of the two stained sections allows to determine precisely the tissue localization and morphology of every biopsy (Fig 4C and 4D), although for some tissues the optical image before LMD allows already to determine the tissue morphology. Table 2 describes the average tissue area cut of each square cut, with a maximum standard deviation of 0.57% that will not significantly impact the sampled tissue area.

### LMD capture efficiency calculated from the compound tissue concentration

The capture efficiency inside a 96-well plate is an important aspect of the workflow. Since the magnification lenses we had during the experiment could not focus on the bottom of the wells of the 96-well plate, we evaluated the capture efficiencies from the calculated loperamide and darunavir concentrations that were sprayed on the tissue section prior to LMD. Thus, after the LMD experiments on 40 μm thick liver tissue sections, the collected biopsies were extracted and analyzed using LC-MS/MS (Table 3). The detection of sprayed darunavir and loperamide in the samples allowed to validate that the tissue cut was collected.

**Table 3:** Capture efficiency after dissection of a whole 96-well template. (A) Capture efficiency of a sprayed fresh frozen liver tissue (20 μm thickness), derived from calculated concentrations of all compounds after LC-MS/MS analysis. (B) Visually checked capture of a fresh frozen brain section (20 μm thickness).

*Template (μm × μm)*	*Tissue losses*	*Capture Efficiency*
***(A) 500 × 500***	13	86%
***270 × 270***	23	76%
***200 × 200***	17	82%
***100 x 100***	8	92%
***50 x 50***	18	81%
***(B) 500 x 500***	7	91%
***270 x 270***	10	89%

For dissected areas larger than 270×270 μm^2^, it was possible to visually check the collected tissue piece in the 96 well plate by eye, and thus, the reported capture efficiencies were determined by visual inspection. Thus, the combination of the visual inspection with the LC-MS/MS analysis for the quantification of the sprayed compounds on the tissue sections, allowed us to assess the collection efficiency for the different 96 squares of the microgrid template. In the case no collected tissue cut was observed in one of the wells, the possibility existed that the successfully dissected tissue was collected in another well. However, the collection of more than one dissected tissue piece did not occur.

Table 3 (S13 File) displays the capture efficiency after dissection of a whole 96 squares microgrid for both sprayed and non-sprayed liver tissue sections. Across all experiments, the average capture efficiency was observed to be 85%. A template of 30×30 μm^2^ squares was also tested but resulted in a measurement below the Lower Limit of Quantification (LLOQ; see Table 4) and was therefore not included in the table. Since the capture of the dissected cut relies purely on gravity and has to drop about half a centimeter into the well of the plate, there are external factors, such as room humidity level and the presence of air flow around the LMD system, that can influence the tissue collection efficiency. Thus, efforts should be made to have a tight control on these parameters, such as monitoring the temperature and humidity in the lab, as well as reducing the airflow where LMD is performed to maximize the capture efficiency.

**Table 4:** Summary of calculated concentrations (ng/mL) of darunavir, loperamide, and an internal JNJ compound with corresponding coefficient of variance in percentage. BQL: Below Quantification Limit.

Template (μm × μm)	*500×500*	*270×270*	*200×200*	*100×100*	*50×50*	*30×30*
***Darunavir (ng/mL)***	30.27 ±18.8%	8.76 ±29.4%	5.22 ±31.7%	1.28 ±32.0%	0.18 ±36.9%	BQL
***Loperamide (ng/mL)***	34.60 ±13.3%	10.01 ±29.5%	6.02 ±30.1%	1.35 ±20.34%	0.21 ±33.01%	BQL
***JNJ compound (ng/mL)***	27.57 ±19.1%	8.14 ±30.3%	4.64 ±30.3%	0.98 ±22.56%	BQL	BQL

In terms of sensitivity, Table 5 (S14 File) displays the calculated LLOQ in μg/g tissue for each compound, based on their LLOQ determined from the calibration curve prepared in the extraction solvent, when using different cut sizes and for a tissue thickness of 40 μm. The calculation is based on the LLOQ expressed in ng/mL of solution that was then normalized to the tissue amount, using the following formula: g tissue = (tissue area) x (tissue thickness) x (average density of liver; [23]), assuming the matrix effect is similar for the different cut sizes and that the extraction yield is 100%. As expected, the sensitivity mainly depends on the surface of tissue dissected. As an example, both loperamide darunavir and the JNJ compound display a calculated LLOQ of 0.9 μg/g tissue when using the templates with single cuts sizes of 500x500 μm^**2**^, while the LLOQ would be 245.3 μg/g tissue for the template with single cuts sizes of 30x30 μm^2^. In addition, the LLOQ of the workflow can be improved using μLC- or nanoLC-MS/MS, at the cost of a lower throughput. Ihn addition, to maximize the sensitivity, consecutive sections can be processed and the cuts at the same location of the tissue can be pooled before analysis.

**Table 5:** Calculated LLOQ for a 40μm thick mouse liver tissue section for the different microgrid templates.

Template (μm × μm)	*500 x 500*	*270 x 270*	*200 x 200*	*100 x 100*	*50 x 50*	*30 x 30*
***Average square area (μm2)***	246363	73483	39969	10134	2563	923
***Darunavir*** ***μg/g***	0.9	3.1	5.7	22.3	88.4	245.3
***Loperamide μg/g***	0.9	3.1	5.7	22.3	88.4	245.3
***JNJ compound μg/g***	0.9	3.1	5.7	22.3	88.4	245.3

## Conclusion

We reported herein the development and implementation of 96 squares microgrids of various sizes, i.e. 30×30 μm^2^ (magnification of 63x), 50×50 μm^2^ (magnification of 63x), 100×100 μm^2^ (magnification of 40x), 200×200 μm^2^ (magnification of 20x), 270×270 μm^2^ (magnification of 10x) and 500×500 μm^2^ (magnification of 5x), for laser microdissection of fresh frozen tissue sections and the direct collection of the cuts in a 96 well plate. The optimization of several parameters, such as tissue thickness, tissue drying time and the selection of the magnification lens for the cuts allowed to optimize the throughput and robustness of the LMD workflow in different tissues (mouse liver and brain). This indiscriminate LMD workflow was developed to be combined with compounds extraction and LC-MS/MS analysis for high-throughput spatial and absolute quantification of drugs in tissue sections. With these templates, 96 sequential tissue cuts can be collected in a time evaluated to be 19 to 32 minutes, depending on the selected cut size, and analyzed in 6.9 h to 7.1 h (collection time + 10 min extraction time + (96 cuts x 4 min run time)). The advantage of using this workflow over classic mass spectrometry imaging relies on the absolute quantification and sensitivity we can achieve.

Moreover, the proposed templates can also be applied for metabolomics and proteomics studies. Next, in view of these current developments in the field of multi-Omics and the increased interest in zooming in on cell specific markers, the developed technique could be a valuable tool to visualize the ‘unseen’, both in a targeted as well as an untargeted workflows.

## Supporting information

S1 File 96 squares microgrid for 30x30 μm cut sizes at a magnification of x63. (SLD)

S2 File 96 squares microgrid for 50x50 μm cut sizes at a magnification of x63. (SLD)

S3 File 96 squares microgrid for 100x100 μm cut sizes at a magnification of x40. (SLD)

S4 File 96 squares microgrid for 200x200 μm cut sizes at a magnification of x20. (SLD)

S5 File 96 squares microgrid for 270x270 μm cut sizes at a magnification of x10. (SLD)

S6 File 96 squares microgrid for 500x500 μm cut sizes at a magnification of x5. (SLD)

S7 File 96 squares microgrid for 30x30 μm cut sizes at a magnification of x63. (TXT)

S8 File 96 squares microgrid for 50x50 μm cut sizes at a magnification of x63. (TXT)

S9 File 96 squares microgrid for 100x100 μm cut sizes at a magnification of x40. (TXT)

S10 File 96 squares microgrid for 200x200 μm cut sizes at a magnification of x20. (TXT)

S11 File 96 squares microgrid for 270x270 μm cut sizes at a magnification of x10. (TXT)

S12 File 96 squares microgrid for 500x500 μm cut sizes at a magnification of x5. (TXT)

S13 File Raw data file of the calculated compounds concentrations in the LMD cuts of the sprayed tissue sections, using the different templates. (XLSX)

S14 File Raw data file of the experimental and calculated LLOQ values of the sprayed compounds on the tissue sections. (XLSX)
